# Combined Phytochemistry and Chemotaxis Assays for Identification and Mechanistic Analysis of Anti-Inflammatory Phytochemicals in *Fallopia japonica*

**DOI:** 10.1371/journal.pone.0027480

**Published:** 2011-11-08

**Authors:** Ming-Yi Shen, Yan-Jun Liu, Ming-Jaw Don, Hsien-Yueh Liu, Zeng-Weng Chen, Clément Mettling, Pierre Corbeau, Chih-Kang Chiang, Yu-Song Jang, Tzu-Hsuan Li, Paul Young, Cicero L. T. Chang, Yea-Lih Lin, Wen-Chin Yang

**Affiliations:** 1 Biotechnology Research Center, Academia Sinica, Taipei, Taiwan; 2 Institute of Pharmacology, National Yang-Ming University, Taipei, Taiwan; 3 National Research Institute of Chinese Medicine, Taipei, Taiwan; 4 Department of Veterinary Medicine, Department of Life Sciences, National Chung Hsing University, Taichung, Taiwan; 5 Institut de Génétique Humaine (CNRS UPR-1142), Montpellier, France; 6 Department of Chinese Medicine, Buddist Tzu Chi General Hospital, Hualien, Taiwan; University of Nebraska Medical Center, United States of America

## Abstract

Plants provide a rich source of lead compounds for a variety of diseases. A novel approach combining phytochemistry and chemotaxis assays was developed and used to identify and study the mechanisms of action of the active compounds in *F. japonica*, a medicinal herb traditionally used to treat inflammation. Based on a bioactivity-guided purification strategy, two anthranoids, emodin and physcion, were identified from *F. japonica*. Spectroscopic techniques were used to characterize its crude extract, fractions and phytochemicals. The crude extract, chloroform fraction, and anthranoids of *F. japonica* significantly inhibited CXCR4-mediated chemotaxis. Mechanistic studies showed that emodin and physcion inhibited chemotaxis via inactivating the MEK/ERK pathway. Moreover, the crude extract and emodin could prevent or treat type 1 diabetes in non-obese diabetic (NOD) mice. This study illustrates the applicability of a combinational approach for the study of anti-inflammatory medicine and shows the potential of *F. japonica* and its anthranoids for anti-inflammatory therapy.

## Introduction

A fundamental feature of inflammation is the migration of leucocytes from blood vessels to inflamed sites. This leukocyte migration, termed chemotaxis, is guided by chemokines and their receptors [1]. Chemotaxis could thus reasonably be a potential target of anti-inflammatory drugs [2]. CXCR4 (fusin) is a CXC chemokine receptor which is expressed in all leukocytes, blastocysts and a variety of cancer cells [3]. On binding to stromal-derived-factor-1 (SDF-1)/CXCL12, its natural ligand, CXCR4 initiates a signaling cascade which includes the activation of focal adhesion kinase (FAK), phosphatidyl inositol 3-kinase (PI3K), extracellular signal-regulated kinase (ERK), Janus kinase (JAK), tyrosine kinase (TYK), phosphatases, nuclear factor kappa B (NFκB) and signal transducer and activator of transcription (STAT). Finally, CXCR4 signaling leads to chemotaxis, locomotion, and adhesion [3].

CXCR4 is an essential gene and its deficiency causes embryonic lethality in mice. It is also involved in the development of autoimmune diseases, HIV infection, angiogenesis, and cancer metastasis [4], [5], [6], [7]. The first FDA approved CXCR4 antagonist, plerixafor/AMD3100, is used to mobilize hematopoietic stem cells, which are collected for use in stem cell graft in patients with hematological cancers. Plerixafor was initially developed to interfere with SDF-1/CXCR4 interaction and shows promise for HIV infection, cancers and autoimmune diseases such as rheumatoid arthritis [8]. However, this drug is expensive because of the difficulty in its total synthesis. There is a demand for the discovery of new CXCR4 antagonists that are at the same time cost-effective and potent.

Phytochemicals and their derivatives have been an extraordinary source of lead compounds and therapeutics in drug development [9]. *F. japonica* (syn: *Polygonum cuspidatum* Sieb. *et* Zucc. or *Reynoutria japonica* Houtt.) is a Polygonaceae plant, which is widespread across Asia and North America. In recent years, this plant has received increasing global attention due to its high resveratrol content [10], [11], [12]. *F. japonica*, also known as Hu-Zhang or Japanese knotweed, is used as Chinese herbal medicine for inflammation, hepatitis, infection, tumors, hypertension, bleeding and hyperlipidemia [13], [14]. It has also been shown to inhibit cholesterol ester synthesis [15] and protein kinases [16]. Stilbenes (resveratrol and piceid) [17] and their glycosides [18] and anthranoids (emodin, physcion, anthraglycoside B, citreorosein, emodin 8-O-β-D-glucopyranoside) [17], [19], [20] and phenolic saccharides [21] have been found in *F. japonica*. Among these, emodin has known cathartic, anti-inflammatory [22], anti-cancer, anti-microbial, diuretic, DNA-binding, and vasorelaxant activities [23], [24], [25]; emodin and physcion showed cytotoxicity against cancer cells [24], [26]; and emodin, citreorosein and emodin 8-O-β-D-glucopyranoside showed phytoestrogen activity [20], [27], [28]. In addition, emodin and physcion appeared to be kinase and tyrosinase inhibitors [16], [29], [30]. Further, anthraglycoside B has been used to treat acute hepatitis and leukocyte reduction [17]; resveratrol and piceid were reported to reduce inflammation, cyclooxygenase and liver injury and have cancer-chemopreventive activities [31]; and phenolic saccharides inhibited DNA primase activity in bacteria [21].

In the present study, a novel approach involving phytochemistry and chemotaxis, was developed to identify the active compounds from medicinal herbs and study their mechanisms of action. The crude extract of *F. japonica*, which has 2 active anthranoid compounds, emodin and physcion, was used to illustrate proof of concept. Based on a bioactivity-directed fractionation and isolation procedure, we evaluated the anti-chemotactic and anti-diabetic activities of the crude extract, chloroform fraction and emodin and/or physcion from *F. japonica*. We further examined the underlying mechanisms by which both anthranoids suppressed chemotaxis. We further showed that *F. japonica* and emodin could prevent or treat the development of type 1 diabetes, an inflammatory disease, in a mouse model. This study demonstrates the feasibility of this novel combinational approach and shows that *F. japonica* and its anthranoids are potential anti-inflammatory agents.

## Materials and Methods

### Ethics statement

All animals were maintained and handled according to the institutional guidelines and the protocol was approved by the Academia Sinica Animal Care and Utilization Committee (protocol number: OMiIBAYW2010043).

### Reagents and cells

WST-1 was purchased from Roche (Mannheim, Germany). Dimethyl sulfoxide (DMSO), methanol, phosphate-buffered saline (PBS), hematoxylin, eosin Y, and resveratrol were purchased from Sigma (MO, USA). Acetonitrile, ethyl acetate and chloroform were purchased from Avantor™ Performance Materials (NJ, USA). SDF-1β, FITC-conjugated anti-CCR5 antibody, anti-CXCR4 antibody, isotype antibody and FITC-conjugated secondary antibody were purchased from R&D Systems (MN, USA). Anti-ERK1/2, anti-phospho-ERK1/2, anti-MEK1/2, and anti-phospho-MEK1/2 were purchased from Cell Signaling (MA, USA). PVDF membrane and ECL immunoblotting detection reagent were purchased from GE healthcare (NJ, USA). Jurkat cells E6.1 (ATCC No. TIB-152), a human T cell line, were grown in RPMI medium as previously published [32]. RPMI 1640 medium was purchased Gibco (CA, USA).

### Preparation and HPLC analysis of crude extract, fractions and anthranoids from *F. japonica*

The root of *F. japonica* was purchased from a local Chinese herbalist and authenticated by one of our authors and a doctor of Chinese medicine, Dr. Chih-Kang Chiang. An *F. japonica* sample (20 g) was ground and extracted with methanol (200 ml × 3) to yield the crude extract. After evaporation, 3.6 g of the crude extract was re-suspended in 540 ml water and partitioned with chloroform (1080 ml × 10), yielding a water fraction and chloroform fraction (621 mg). The water fraction was partitioned with ethyl acetate (720 ml × 5), resulting in a water fraction (1554 mg) and an ethyl acetate fraction (1225 mg). The high performance liquid chromatography (HPLC) profile of the crude extracts and fractions was constructed using a RP-18 column [Phenomenex Luna 5 µ C18 (2), 250×4.6 mm] at a flow rate of 0.5 ml/min, detected at ultraviolet (UV) 254 nm. The solvents used for HPLC were: (A) water with 0.05% TFA and (B) acetonitrile with 0.05% TFA. The solvent gradients for HPLC were set as follows: (1) 10% B to 50% B from 0 min to 50 min, (2) 50% B to 90% B from 50 min to 100 min, (3) 90% B to 100% B from 100 min to 110 min, (4) 100% B to 100% B from 110 min to 135 min. Emodin (100 mg) and physcion (40 mg) from the active chloroform fraction of *F. japonica* and resveratrol from the crude extract were identified by comparing their nuclear magnetic resonance (NMR), UV and/or mass spectrometry (MS) data with previously reported data [17], [33], [34]. The purity of the three compounds was over 95% as determined by ^1^H-NMR and HPLC purification.

### Chemotaxis and WST-1 assays

Jurkat cells were pre-treated with crude extract, fractions or anthranoid compounds for the indicated time. The cells were then transferred into transwell inserts with a pore size of 5 µm. Transwell inserts containing the cells were put into a 24-well plate where SDF-1β or vehicle (PBS) were added to the RPMI medium. After 4 hours, the cells that migrated to the bottom of the 24-well plate were quantified using hemocytometry. The migration index (MI) was defined by the following formula: MI (%)  =  100 × (number of drug-treated cells migrating toward SDF-1β minus number of drug-treated cells migrating toward PBS)/(number of vehicle-treated cells migrating toward SDF-1β minus number of vehicle-treated cells migrating toward PBS). For WST-1 assays, Jurkat cells (5×10^3^) were incubated with crude extract, fractions and anthranoid compounds for 24 hours. After extensive washing, the cells were incubated with WST-1 for 1 hour and measured at 440 nm using an enzyme-linked immunosorbent assay reader.

### FACS analysis

Jurkat cells were pre-treated with DMSO vehicle, emodin (1 µg/ml) or physcion (1 µg/ml) for 1 hour. After washing, the cells were stained with isotype antibody or anti-CXCR4 antibody plus FITC-conjugated secondary antibody. The cells were subjected to FACS analysis and results were analyzed using FCS Express software.

### Western blot

Jurkat cells were pre-treated with DMSO vehicle, emodin (1 µg/ml) and physcion (1 µg/ml) for 1 hour. The cells were stimulated with SDF-1β for 0, 5, 10 and 15 minutes. Total lysates of each sample were subjected to SDS-PAGE and blotted with the antibodies against ERK1/2 or their phosphorylated forms. After stripping, the same membrane was re-blotted with MEK1/2 and their phosphorylated forms. Proteins were visualized using ECL kits and detected using ChemiGenius image analysis system (Syngene, Cambridge, UK). The relative intensities of the protein bands were quantitated using Syngene GeneTools software.

### Drug administration, diabetes measurement and histological examination

Female NOD mice were intraperitoneally administered with 0.2 ml of PBS, the crude extract (40 mg/kg body weight (BW)) or emodin (4, 20 and 40 mg/kg BW) of *F. japonica*, three times per week, from 4 to 30 weeks of age (prevention model), and the crude extract (40 mg/kg) of *F. japonica*, three times per week, from 9 to 30 weeks of age (treatment model) unless indicated otherwise. The above mice were monitored every week for glycosuria and glycemia using a Clinistix strip (Bayer) and an Elite glucometer (Bayer), respectively. Animals with 2 consecutive measurements of blood glucose over 250 mg/dl were considered diabetic. The pancreata of NOD mice, aged 12 weeks (except when indicated otherwise), were embedded in wax, cut into 5-µm sections and stained with hematoxylin and eosin. Over 20 islets per pancreas were examined to determine the severity of insulitis. The number of islets in each category is presented as a percentage of total islets observed. Scoring categories were: 0, no insulitis; 1, leukocyte infiltration around the islets (peri-insulitis); 2, <50% leukocyte infiltration (moderate); and 3, >50% leukocyte infiltration (severe insulitis) [35].

### Statistical analysis

Data from three experiments or more are presented as mean ± standard error (SE). For diabetic incidence, the log-rank test was used to determine if a group was statistically significant from the control group. For the other experiments, ANOVA was performed to determine whether there was a significant difference between treatment groups and control groups. P values of less than 0.05 were considered statistically significant.

## Results

### Extraction, partition, isolation and identification of two anthranoid compounds from *F. japonica* by bioactivity-guided fractionation and isolation

**Figure 1:**
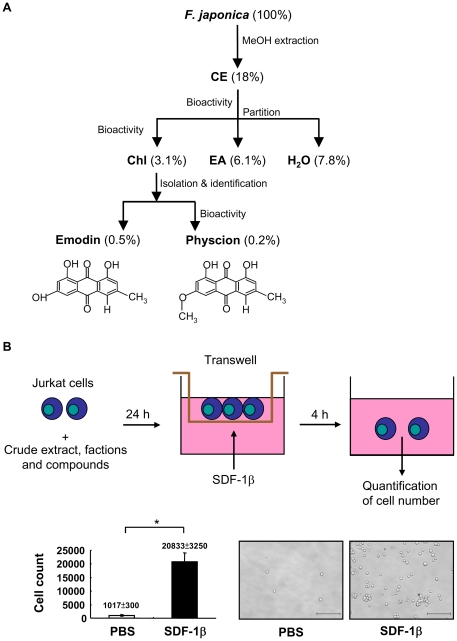
Bioactivity-guided fractionation and isolation strategy involving extraction, fractionation, purification and chemotaxis assays. A. *F. japonica* root was ground and then extracted with methanol. The crude extract (CE) was reconstituted with water and its bioactivity tested using chemotaxis assay. The same crude extract was sequentially partitioned with chloroform (Chl), ethyl acetate (EA) and water (H_2_O). After reconstitution, the bioactivity of the 3 fractions was further tested. Finally, active compounds, emodin and physcion, were purified from the active chloroform fraction. The dry weights of the crude extract, fractions and phytochemicals of *F. japonica* are indicated as percentages of the dry weight of the raw plant material. B. Chemotaxis assays were used to evaluate the bioactivity of the crude extract, fractions and phytochemicals of *F. japonica*. Jurkat cells were used as a bioassay platform to test SDF-1β-mediated migration (upper panel). The cell number of Jurkat cells migrating in response to PBS vehicle or SDF-1β was counted. Data from 3 independent experiments are presented as means ± SE (lower panel). Representative pictures are shown. Scale bar, 200 µm.

**Figure 2:**
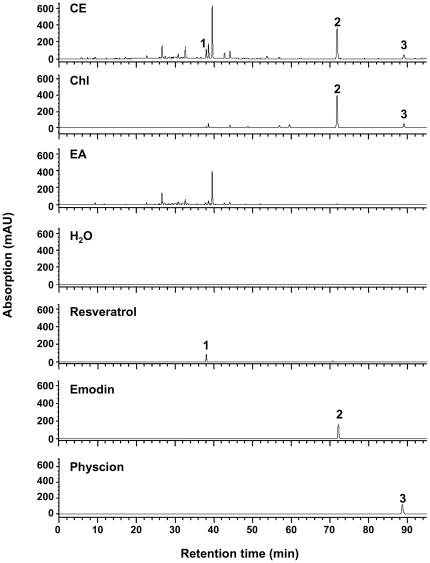
HPLC profiles of crude extracts, fractions and compounds present in *F. japonica*. Methanol crude extract (CE), water fraction (H_2_O), ethyl acetate (EA) fraction, chloroform (Chl) fraction, and compounds (resveratrol, emodin and physcion) from *F. Japonica* were analyzed using a RP-18 HPLC column and detected with a UV detector at 254 nm. Peaks **1** (resveratrol), **2** (emodin) and **3** (physcion) are shown.

A novel combinational chemotaxis assay-guided fractionation and isolation method was used to evaluate the anti-inflammatory properties of the medicinal herb *F. japonica* (Figure 1) as a means to test the general feasibility of the protocol for the evaluation of the active compounds in medicinal herbs. Briefly, the root of *F. japonica* was ground, and extracted with methanol to yield the crude extract. The methanol crude extract was reconstituted with water and sequentially partitioned with chloroform and ethyl acetate, yielding chloroform, ethyl acetate and water fractions. Finally, two active anthranoid compounds, emodin and physcion, and an index compound, resveratrol, were isolated and identified using nuclear magnetic resonance (NMR), mass spectrometry (MS) and ultraviolet (UV) methods. NMR spectra of the compounds are shown below. Emodin: ^1^H NMR (500 MHz, pyridine-d_5_) *δ*: 12.54, 12.44 (br s, 2H, 1- and 8- OH), 7.72 (s, 1H, H-4), 7.70 (d, J = 2.2 Hz, 1H, H-5), 7.14 (s, 1H, H-7), 6.99 (d, J = 2.2 Hz, 1H, H-2), 5.01 (1H, s, 3-OH), 2.24 (s, 3H, CH_3_). ^13^C NMR (500 MHz, DMSO) *δ*: 164.4 (C-1), 120.4 (C-2), 148.2 (C-3), 124.1 (C-4), 108.8 (C-5), 161.4 (C-6), 107.9 (C-7), 165.7 (C-8), 189.6 (C-9), 181.4 (C-10), 132.8 (C-4a), 108.8 (C-8a), 113.3 (C-9a), 135.1 (C-10a), 21.5 (–CH3). Pyscion: ^1^H NMR (500 MHz, CDCl_3_) *δ*: 12.30, 12.10 (s, 2H, 1- and 8-OH), 7.62 (s, 1H, H-5), 7.36 (d, J = 2.3 Hz, 1H, H-4), 7.07 (s, 1H, H-7), 6.67 (d, J = 2.3 Hz, 1H, H-2), 3.92 (s, 3H, OCH_3_), 2.43 (s, 3H, CH_3_). ^13^C NMR (500 MHz, CDCl_3_) *δ*: 165.2 (C-1), 106.8 (C-2), 166.6 (C-3), 108.2 (C-4), 121.3 (C-5), 148.5 (C-6), 124.5 (C-7), 162.5 (C-8), 190.8 (C-9), 182.1 (C-10), 133.2 (C-11), 113.7 (C-12), 110.2 (C-13), 135.2 (C-14), 22.2 (C-15), 56.1 (OCH_3_). Resveratrol: ^1^H NMR (500 MHz, acetone-d_6_)*δ*: 8.45 (s, 1H, 4′-OH), 8.18 (s, 2H, 3- and 5-OH), 7.42 (d, J = 8.6 Hz, 2H, H-3′ and -5′), 7.02 (d, J = 16.2 Hz, 1H, β), 6.88 (d, J = 16.2 Hz, 1H, α), 6.54 (d, J = 2.2 Hz, 2H, H-2 and -6), 6.35 (d, J = 8.6 Hz, 2H, H-2′ and -6′), 6.26 (t, J = 2.2 Hz, 1H, H-4). ^13^C NMR (500 MHz, acetone-d_6_) *δ*: 141.0 (C-1), 105.7 (C-2), 159.7 (C-3), 102.7 (C-4), 159.7 (C-5), 105.7 (C-6), 130.0 (C-1′), 128.8 (C-2′), 116.5 (C-3′), 158.2 (C-4′), 116.4 (C-5′), 128.8 (C-6′), 126.9 (α), 129.2 (β). Our NMR data for emodin, physcion and resveratrol were identical to those published elsewhere [17], [33], [34]. UV and MS data also confirmed the identity of emodin, physcion and resveratrol (Figures S1 and S2). The crude extract, water fraction, ethyl acetate fraction, chloroform fractions, and compounds of *F. japonica* were profiled using high performance liquid chromatography (HPLC) (Figure 2). The presence of emodin, physcion and/or resveratrol in the crude extract and chloroform fraction was then further confirmed using UV and MS analysis (Figures S1 and S2).

### Effect of crude extract, fractions and anthranoid compounds of *F. japonica* on CXCR4-mediated chemotaxis

Since CXCR4 is expressed in all leukocytes, CXCR4 was selected as the target molecule in chemotaxis assays. First, we set up a chemotaxis assay in Jurkat cells, a human leukemia T-cell line. We found that SDF-1β, the CXCR4 ligand, could induce a 20-fold increase in T-cell migration as compared to vehicle control (lower panel, Figure 1B).

**Figure 3:**
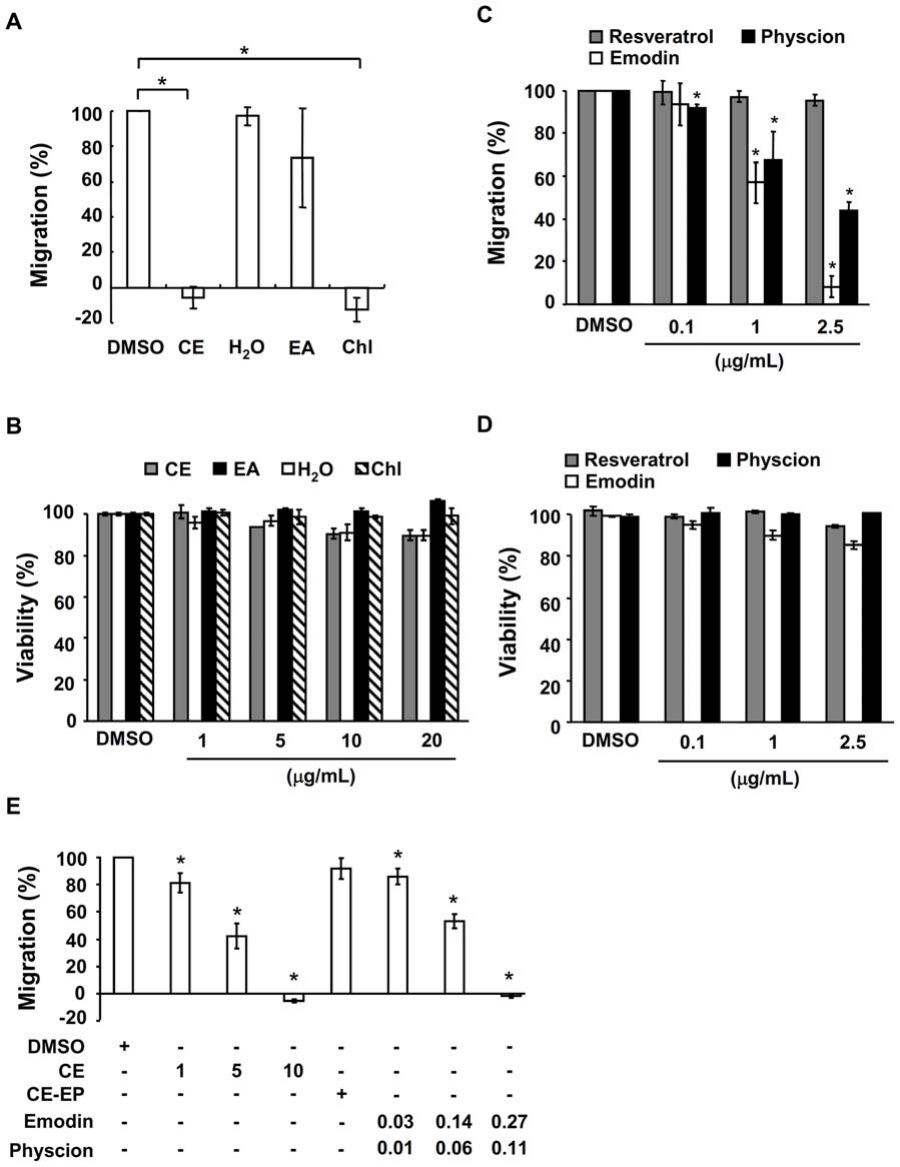
Effect of crude extract, fractions and compounds of *F. japonica* on chemotaxis and cell viability. A. Jurkat cells were pre-treated with vehicle (DMSO) and the crude extract (CE), chloroform (Chl) fraction, ethylacetate (EA) and water fraction (H_2_O) of *F. japonica* at 10 µg/ml for 24 h. The cells were treated with PBS and SDF-1β, respectively, for an additional 4 h in transwell microplates. The number of cells in the bottom well were counted. Cell migration is indicated as migration index (%), as defined in Materials and Methods. B. Jurkat cells were pre-treated the same way as described for Figure 3A, and cell viability was measured using WST-1 test. C. Jurkat cells underwent the same procedure as Figure 3A except that they were pre-treated with DMSO vehicle, resveratrol, emodin and physcion at 0.1, 1 and 2.5 µg/ml for 24 h. D. Jurkat cells were pre-treated the same way as described for Figure 3B and cell viability was measured using WST-1 test. E. Jurkat cells underwent the same procedure as Figure 3A except that DMSO vehicle, the crude extract (CE, 1, 5 and 10 µg/ml), the crude extract without emodin and physcion (CE-EP, 9.62 µg/ml) and a mixture of emodin (0.03, 0.14 and 0.27 µg/ml) and physcion (0.01, 0.06 and 0.11 µg/ml) of *F. japonica* were used. Data from 3 independent experiments are expressed as mean ± SE. *P*<0.05 (*).

We then pre-treated Jurkat cells with the crude extract, fractions and compounds of *F. japonica* for 24 hours and tested the chemotactic response of cells to SDF-1β. We found that the crude extract and chloroform fraction of *F. japonica* at 10 µg/ml completely inhibited CXCR4-mediated chemotaxis in Jurkat cells (Figure 3A). However, the ethyl acetate and water fractions did not show any significant inhibition (Figure 3A). Based on bioactivity-guided fractionation and isolation, two active compounds, emodin and physcion, were isolated and identified from the most active chloroform fraction (Figure 2, S1 and S2). Resveratrol present in the crude extract of *F. japonica* was used as an index compound. This index compound did not inhibit CXCR4-mediated chemotaxis (Figure 3C). In sharp contrast, for the first time, emodin and physcion significantly suppressed CXCR4-mediated chemotaxis in a dose-dependent manner (Figure 3C). The half maximal inhibitory concentrations (IC_50_) of the crude extract, chloroform fraction, emodin and physcion of *F. japonica* were 4.5, 3.8, 1.3 and 2.1 µg/ml, respectively. The viability of cells exposed to a dose of methanol crude extract, the 3 fractions or the anthranoids of *F. japonica* used in this study for 24 hours was over 90% based on WST-1 assay (Figures 3B and 3D). The data suggest that the suppression of chemotaxis by the crude extracts, 3 fractions and anthranoids in this study was not due to cytotoxicity.

To assess whether emodin and physcion were, in fact, the main active compounds in the crude extract of *F. japonica*, we compared the anti-chemotactic activities of the crude extract of *F. japonica* (10 µg/ml), the crude extract of *F. japonica* depleted of emodin and physcion (9.62 µg/ml), a mixture of emodin (0.27 µg/ml) and physcion (0.11 µg/ml) in the same ratio and quantity as both in the crude extract, a mixture of emodin (0.14 µg/ml) and physcion (0.06 µg/ml), and a mixture of emodin (0.03 µg/ml) and physcion (0.01 µg/ml). We found that the crude extract of *F. japonica* showed similar anti-chemotactic activity to the reconstituted mixture of emodin and physcion (Figure 3E). Conversely, the crude extract of *F. japonica* lacking both emodin and physcion lost its anti-chemotactic activity (Figure 3E). Overall the data suggest that emodin and physcion are the major active compounds in the crude extract of *F. japonica* that result in inhibition of CXCR4-mediated chemotaxis, although the existence of other minor active compounds can not be ruled out.

### Mechanistic study of emodin and physcion in CXCR4-mediated chemotaxis

**Figure 4:**
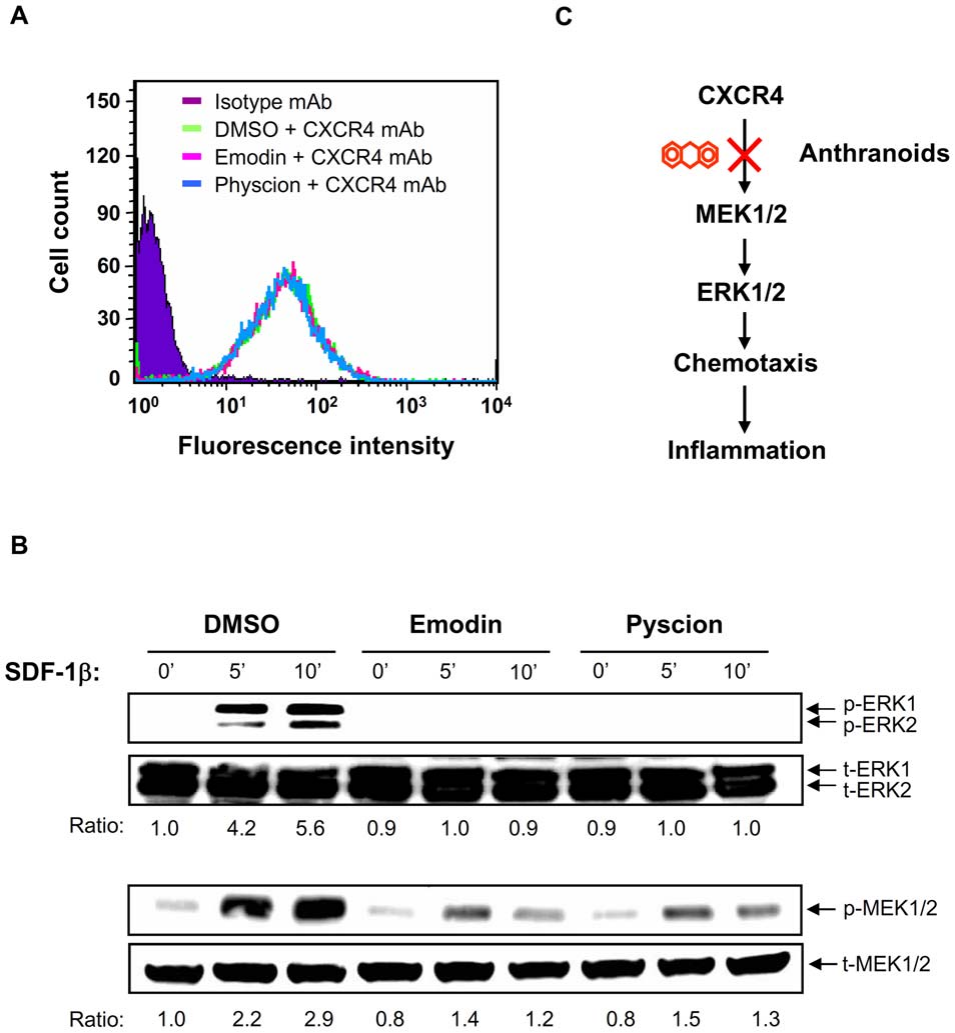
Effect of emodin and physcion on CXCR4 signaling. A. Jurkat cells were incubated with emodin (1 µg/ml), physcion (1 µg/ml) or DMSO vehicle for 1 h at 37°C. After extensive washing, the cells were stained with anti-CXCR4 antibody or isotype antibody (control), followed by FITC-conjugated secondary antibody. The surface expression level of CXCR4 is shown as logarithmic fluorescence intensity. Data are representative examples of three independent experiments. B. The cells, with the same pre-treatment as Figure 4A, were stimulated with SDF-1 (100 ng/ml) from 0 to 15 min. Total lysates were analyzed by Western blot with antibody against ERK1/2 (t-ERK1/2) and their phosphorylated proteins (p-ERK1/2) (upper panel). After stripping, the same membrane was re-blotted with the antibody against MEK (t-MEK1/2) and their phosphorylated proteins (p-MEK1/2) (lower panel). The ratio was obtained by normalizing the signal of the phosphorylated protein to that of the total protein. C. Working model showing that the anthranoids, emodin and physcion, inhibit CXCR4-implicated chemotaxis and, in turn, inflammation.

Inhibition of chemotaxis by emodin and physcion could result from a decrease in the surface expression of CXCR4 receptors. To rule out this possibility, both anthranoids were used to treat Jurkat cells. After one hour, the CXCR4 expression level in Jurkat cells was measured using fluorescence-activated cell sorting (FACS) analysis. No difference was observed in the level of expression of CXCR4 on the cell surface (Figure 4A) in treated and control cells, suggesting that emodin and physcion do not act at the chemokine receptor level.

We subsequently checked the signaling molecules downstream of the CXCR4 receptor. Mitogen-activated protein kinases (MAPKs) are known to function downstream of chemokine receptors [36]. Therefore, we checked whether emodin and physcion affected ERK1/2 kinases in the CXCR4 pathway. Immunoblot data showed that both compounds inhibited the phosphorylation of ERK1/2 kinase triggered by SDF-1β (Figure 4B). Furthermore, we examined the effect of emodin and physcion on MAPK kinase (MEK)1/2, an upstream activator of ERK1/2. Both anthranoids suppressed the phosphorylation of MEK1/2. These data suggest that emodin and physcion in *F. japonica* suppress CXCR4-mediated chemotaxis and subsequently inflammation, via inactivation of the MEK/ERK signaling cascade (Figure 4C).

### Effect of crude extract and emodin from *F. japonica* on type 1 diabetes

**Figure 5:**
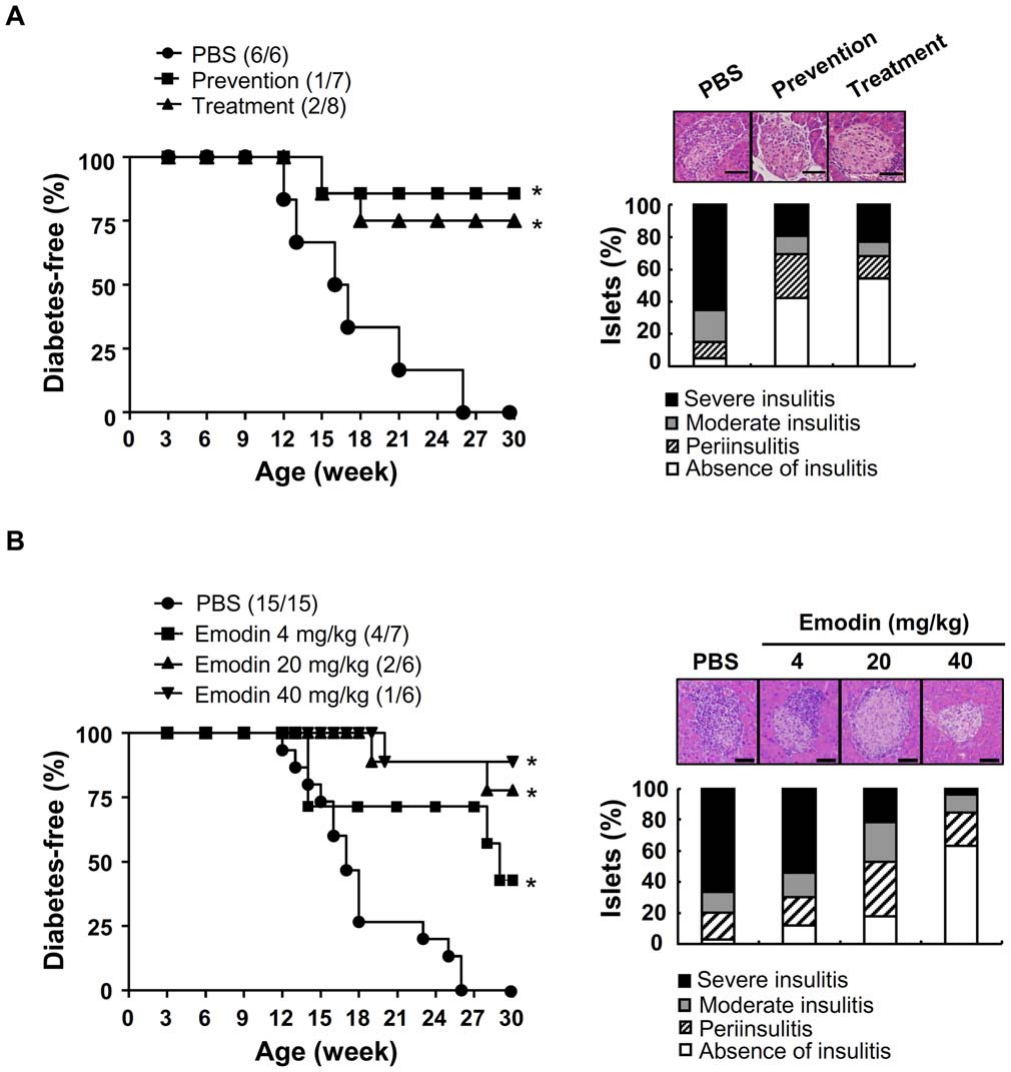
Effect of crude extract and emodin of *F. japonica* on diabetes in NOD mice. A. NOD mice were administered PBS control, crude extract of *F. japonica* at 40 mg/kg, 3 times per week, from 4 to 30 weeks (prevention), or the crude extract of *F. japonica* at 40 mg/kg, 3 times per week, from 9 to 30 weeks (treatment). The cumulative incidence of diabetes in mice, from birth to 30 weeks of age, was monitored (left panel). Mouse pancreata were stained with hematoxylin and eosin (right panel). The severity of insulitis in the NOD pancreata was scored as described in Materials and Methods and plotted as stacked histograms (right panel). B. NOD mice underwent the same procedure as described for Figure 5A except that they received PBS control or emodin (4, 20 and 40 mg/kg), 3 times per week, from 4 to 30 weeks (left panel). Mouse pancreata were stained with hematoxylin and eosin (right panel). The number of diabetic mice (numerator) and total number of mice (denominator) in each group is indicated in the parentheses. *P*<0.05 was considered to be statistically significant (*). Scale bar, 100 µm.

NOD mice spontaneously develop insulitis at the age of 4 to 6 weeks and diabetes at the age of 9 weeks and beyond. To further evaluate the prophylactic and therapeutic effect of the crude extract of *F. japonica* on type 1 diabetes, we administered crude extract of *F. japonica* to NOD mice from 4 to 30 weeks of age (prevention model), and from 9 to 30 weeks of age (treatment model). All NOD mice (100%) treated with vehicle developed type 1 diabetes from the age of 26 weeks (left panel, Figure 5A). In contrast, the crude extract of *F. japonica* had reduced diabetes by 86% in the prevention model group, and by 75% in the treatment model group at 30 weeks of age (left panel, Figure 5A). We also examined the effect of the crude extract on insulitis in 12-week-old NOD mice, as measured by leukocyte infiltration and islet architecture in pancreatic islets. Preventative-treatment or treatment after disease onset with the crude extract of *F. japonica* dramatically decreased leukocyte infiltration into the islets and destruction of pancreatic islets (right panel, Figure 5A). Next, we further tested the prophylactic effect of active anthranoids on type 1 diabetes. Due to a limit in the quantity of physcion available, we only tested the prophylactic effect of emodin, the most active anthranoid in the study, on type 1 diabetes. Like the crude extract of *F. japonica*, emodin dose-dependently prevented type 1 diabetes in NOD mice as measured by the reduction in incidence of diabetes (left panel, Figure 5B), leukocyte infiltration into pancreatic islets and islet destruction (right panel, Figure 5B).

Overall, the animal data suggest that the crude extract of *F. japonica* and emodin suppress type 1 diabetes.

## Discussion

Spectroscopic methods and chemotaxis have been successfully used individually in the pharmaceutical industry. However, to our knowledge the feasibility of combining both methods has not been evaluated, and realization and validation of such a combinational approach has not been attempted. In this study, these methods were combined and evaluated as a novel system for the identification and mechanistic study of the active anthranoids from *F. japonica* (Figures 1, 2, 3 and 4). The chemotaxis-aided approach proved to be simple, straightforward, and highly sensitive. *F. japonica* has been previously reported to contain emodin and physcion [34]. Moreover, emodin and *F. japonica* were shown to be the inhibitors of tyrosine and serine/threonine kinases [16], [30], [37], [38]. We, for the first time, showed that two active compounds, emodin and physcion, present in *F. japonica* plant, could inhibit CXCR4-mediated chemotaxis in a MEK/MAPK-dependent fashion. More importantly, this proof-of-concept study suggests that this combinational approach could be broadly and efficiently applied for research and development of anti-inflammatory agents mined from natural sources and studies in medicinal chemistry.

Interference with chemokine function is a promising inflammatory therapy [2]. The most well-known examples of chemokine receptor antagonists are plerixafor, maraviroc and vicriviroc [8], [39]. Plerixafor is a CXCR4 receptor antagonist which assists in the mobilization of hematopoietic stem cells, whilst maraviroc and vicriviroc are CCR5 receptor antagonists for the treatment of HIV infection. These chemokine receptor antagonists have shown potent anti-inflammatory effects on different immune disorders in different models [8], [40]. In addition to receptor antagonists, it is imaginable that targeting cytoplasmic and nuclear molecules downstream of the chemokine receptors is an alternative approach to block the chemokine signaling.

Chemokine receptors are G protein-coupled receptors. Upon binding to chemokines, these receptors activate G proteins, tyrosine kinases, serine/threonine kinases and phospholipase [36], [41]. Emodin and *F. japonica* have previously been shown to be inhibitors of tyrosine and serine/threonine kinases [16], [30], [37], [38]. Consistent with these previous publications, our results showed that emodin and physcion, isolated from *F. japonica*, inhibited the activation of MEK/ERK kinases, a serine/threonine kinase family, mediated by CXCR4 in T-cells (Figure 4B). However, neither of the two anthranoid compounds affected the expression level of CXCR4 receptor (Figure 4A). These findings suggest that unlike the receptor antagonists, these anthranoids target the intraceullar proteins downstream of the chemokine receptors and may be used as alternative inhibitors of chemokine signaling. MAPKs are known to modulate inflammatory responses and are thought to be attractive molecular targets for anti-inflammatory therapy [42]. The demonstration of inactivation of MEK/ERK pathway by both these antharnoids makes them extremely interesting potential natural anti-inflammatory therapies.

Inflammation is a complex physiological process associated with a variety of diseases. Research and development of effective anti-inflammatory agents with distinct mechanisms of action is important to cure these diseases. *F. japonica* is known as a medicinal herb for inflammation. Two recent studies showed that the extracts of *F. japonica* containing a relatively low dose of resveratrol had anti-oxidant and anti-inflammatory effects in human [43], [44]. These observations raised a possibility that some compounds rather than resveratrol in the extracts of *F. japonica* also had anti-inflammatory activity. Here, we showed that anthranoids present in the *F. japonica* extract had anti-inflammatory activity by inhibiting leukocyte migration (Figure 3). Besides, anthranoids exhibit a variety of bioactivities, some of which are used clinically. Sennosides, anthranoids derived from the leaves of plants from the *Senna* genus, are currently sold as over-the-counter laxatives for constipation [45]. Emodin and physcion have been proposed as anti-tumor agents [24], [26] and emodin was used to treat hepatitis via inhibition of NFκB activation [22]. The fact that *F. japonica* crude extract, and its anthranoids suppress CXCR4-mediated leukocyte migration further supports their traditional use as an anti-inflammatory medication. Furthermore, we showed, for the first time, that *F. japonica* and its most active compound, emodin, could prevent or treat the development of type 1 diabetes, an inflammatory disease, in a mouse model (Figure 5).

In summary, we combined phytochemical and chemotaxis techniques to into a platform suitable for the identification and study of the mechanisms of action of active compounds in medicinal herbs used to treat inflammation. We showed that *F. japonica* and its active compounds, emodin and physcion, suppress inflammation via inhibition of leukocyte chemotaxis. Therefore, *F. japonica* and emodin prevent islet destruction and type 1 diabetes in NOD mice. Mechanistic study showed that both the anthranoids and *F. japonica* inhibit CXCR4-implicated leukocyte migration via inactivation of MEK/ERK pathway downstream of the CXCR4 receptor. These findings support the claims of the benefits of *F. japonica* in the traditional Chinese herbal medicine, and suggest that *F. japonica*, may be of use in the treatment of type 1 diabetes. In addition, the data on the active constituents, emodin and physcion, suggest a novel pharmaceutical use for these anthranoids for inflammation.

## Supporting Information

Figure S1 **UV spectra of anthranoids and resveratrol present in the** ***F. japonica*** **crude extract and fraction.** The crude extract and chloroform fraction of *F. japonica* and their standard compounds (resveratrol, emodin and physcion) were subjected to high performance liquid chromatography and detected with a diode array detector at 254 nm as described in the Materials and Methods section. The UV spectra of peaks **1** (resveratrol), **2** (emodin) and **3** (resveratrol) are indicated. Peaks **1** to **3** correspond to the same peaks as Figure 2.
(TIF)

Figure S2 **Mass spectra of anthranoids and resveratrol present in the** ***F. japonica*** **crude extract and fraction.** The crude extract and chloroform fraction of *F. japonica* and 3 standard compounds (resveratrol, emodin and physcion) were subjected to HPLC-ESI-MS. The MS scans were performed in negative ion mode (m/z 200 to m/z 500). Peaks **1** (37.6 min), **2** (72.1 min), and **3** (88.9 min) of the crude extract showed ion signals at m/z 227, m/z 269 and m/z 283, respectively (upper row). Peaks **2** (71.9 min) and **3** (89.0 min) of the chloroform fraction showed ion signals at m/z 269 and m/z 283, respectively (middle row). Peaks **1 (**resveratrol, 37.4 min), **2** (emodin, 71.9 min), and **3** (physcion, 89.2 min) showed ion signals at m/z 227, m/z 269 and m/z 283, respectively (lower row). The retention time is indicated in the parentheses. Peaks **1** to **3** correspond to the same peaks as Figure 2.
(TIF)
