# Correction: Combined Phytochemistry and Chemotaxis Assays for Identification and Mechanistic Analysis of Anti-Inflammatory Phytochemicals in *Fallopia japonica*

**DOI:** 10.1371/annotation/e81927cf-e940-4604-acfa-44b5655e40a3

**Published:** 2012-06-01

**Authors:** Ming-Yi Shen, Yan-Jun Liu, Ming-Jaw Don, Hsien-Yueh Liu, Zeng-Weng Chen, Clément Mettling, Pierre Corbeau, Chih-Kang Chiang, Yu-Song Jang, Tzu-Hsuan Li, Paul Young, Cicero L. T. Chang, Yea-Lih Lin, Wen-Chin Yang

The affiliations of the fourteenth author, Wen-Chin Yang, are incorrect. Wen-Chin Yang is affiliated with 1,2, and the Biotechnology Research Center, Academia Sinica and Department of Life Sciences, National Chung Hsing University, Taichung, Taiwan.

