# Supplementary figures and images for: Combined Phytochemistry and Chemotaxis Assays for Identification and Mechanistic Analysis of Anti-Inflammatory Phytochemicals in Fallopia japonica
Source: PLoS One. 2011 Nov 8;6(11):e27480. doi: 10.1371/journal.pone.0027480 (PMC3210798; doi:10.1371/journal.pone.0027480)

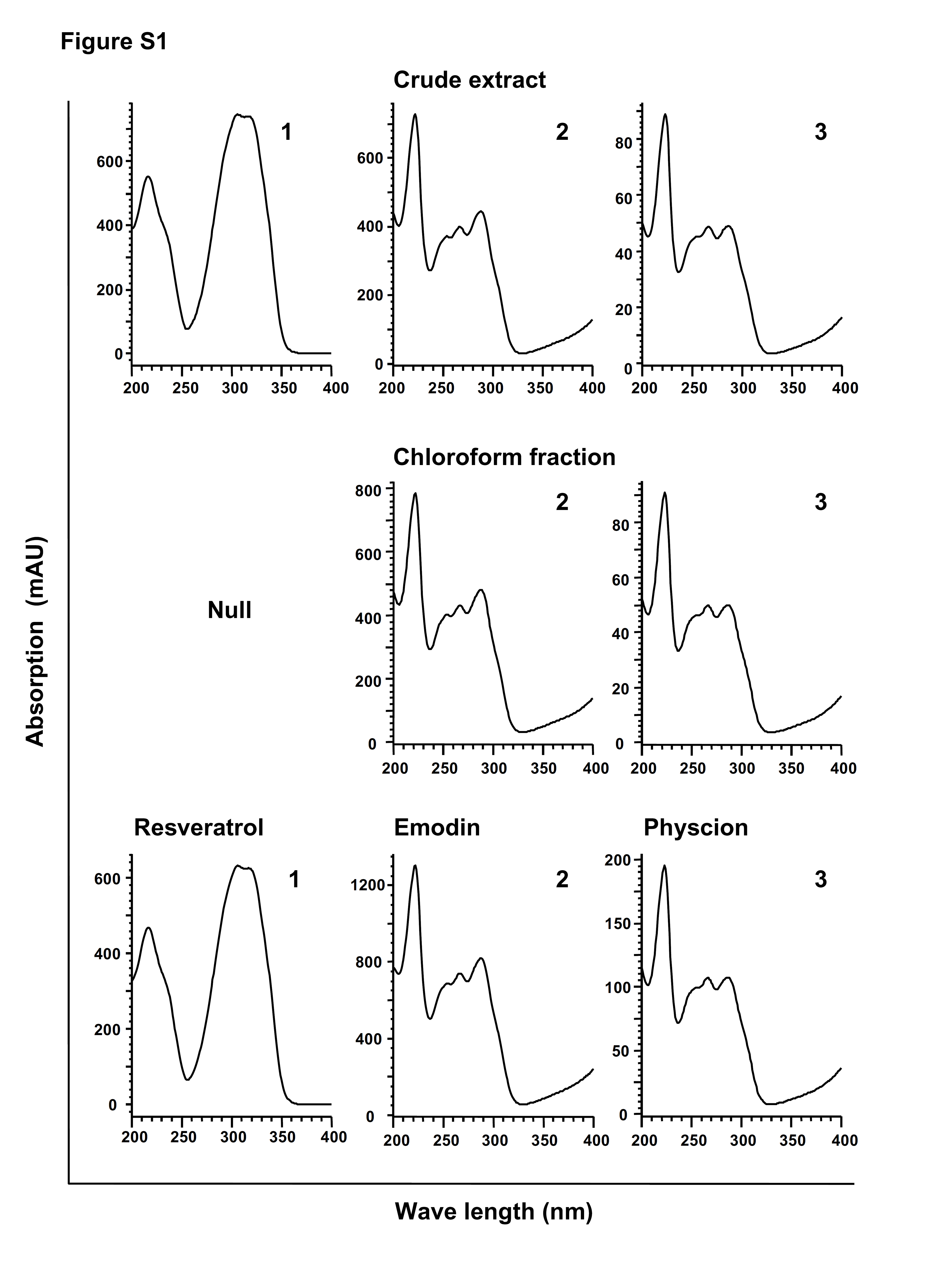

Supplement: Figure S1 — UV spectra of anthranoids and resveratrol present in the F. japonica crude extract and fraction. The crude extract and chloroform fraction of F. japonica and their standard compounds (resveratrol, emodin and physcion) were subjected to high performance liquid chromatography and detected with a diode array detector at 254 nm as described in the Materials and Methods section. The UV spectra of peaks 1 (resveratrol), 2 (emodin) and 3 (resveratrol) are indicated. Peaks 1 to 3 correspond to the same peaks as Figure 2. (TIF) [file pone.0027480.s001.tif]
